# Andexanet alfa Reduces Hematoma Expansion Following Controlled Cortical Impact in Mice Pretreated with Rivaroxaban

**DOI:** 10.1007/s12028-026-02463-w

**Published:** 2026-03-03

**Authors:** Franziska Lieschke, Sarah Gelhard, Michelle Rosenthal-Rueckeis, Christian Grefkes, Ferdinand O. Bohmann

**Affiliations:** 1 Department of Neurology, University Hospital, Goethe University Frankfurt, Theodor-Stern-Kai 7, 60596 Frankfurt am Main, Germany; 2 Department of Neurology with Experimental Neurology, Charité Universitätsmedizin Berlin, Berlin, Germany

**Keywords:** Traumatic brain injury, Anticoagulation, Direct oral anticoagulant, Secondary injury, Factor Xa inhibitor reversal, Trauma, Experimental model, Intracerebral hemorrhage

## Abstract

**Objective:**

With the introduction of andexanet alfa, a specific antidote is now available to address life-threatening bleeding associated with factor Xa inhibitors. In this study, we explore its use in an experimental model of traumatic brain injury (TBI), mimicking a closed head trauma under rivaroxaban-induced anticoagulation.

**Methods:**

Male C57BL6 mice were fed with rivaroxaban (10 mg/kg body weight). Subsequently, TBI was induced by controlled cortical impact (CCI) and andexanet alfa or placebo were administered as intravenous bolus injections. Edema and hemorrhage volume was quantified by magnetic resonance imaging (MRI) 24 h and 7 days after CCI. Functional outcome was assessed at day 1, 3, and 7 thereafter.

**Results:**

Andexanet alfa led to reduced hemorrhage volume 24 h and 7 days after CCI as compared with control group without reversal of anticoagulation (2.9 ± 1.4 µl vs. 5.2 ± 3.3 µl, *p* = 0.02; 3.4 µl ± 1.5 µl vs. 5.5 µl ± 2.4 µl, *p* = 0.04). Along with the smaller hematoma sizes in the MRI, edema volume was significantly lower in mice treated with andexanet alfa 24 h and 7 days after CCI (−6.3% of contralateral hemisphere, *p* = 0.0002; and −7.1% of contralateral hemisphere, *p* = 0.006). While functional outcomes did not differ at 24 h following TBI, andexanet alfa improved neurological deficits after 7 days.

**Conclusions:**

Our experimental data suggests that the use of andexanet alfa improves functional outcomes by reduction of factor Xa inhibitor–associated hematoma expansion in the subacute phase following TBI.

**Supplementary Information:**

The online version contains supplementary material available at 10.1007/s12028-026-02463-w.

## Introduction

Traumatic brain injury (TBI) is a leading global cause of death and disability, with its prevalence rising [1–4]. With an aging population, traumatic brain injury (TBI) is increasingly affecting elderly individuals [1, 5], many of whom are treated with anticoagulants. The incidence of intracranial hemorrhage after mild TBI in anticoagulated patients remains clinically relevant, with reported rates of 6.4% in patients using direct oral anticoagulants [6, 7]. Even after mild TBI, this represents a considerable risk, providing a key rationale for conducting the present study [6, 8].

Current therapeutic strategies focus on managing progressive secondary hemorrhage and ischemic injury, complications that occur in up to 70% of TBI cases [9–13]. Key measures include optimizing coagulation (e.g., reversal therapies for anticoagulation-associated hemorrhages), maintaining consistent blood and cerebral pressure, and, when necessary, employing surgical interventions. These approaches are crucial, as the progression of hemorrhagic contusions strongly correlates with poor clinical outcomes [14–16]. Both anticoagulant use and injury mechanisms add complexity to treatment and prognosis. In TBI with consecutive hemorrhage, anticoagulation exacerbates bleeding and secondary damage through inflammatory cascades, blood–brain barrier disruption, and edema [17]. Thus, reversing anticoagulation is crucial. With the introduction of andexanet alfa, a specific antidote is now available to address life-threatening bleeding associated with factor Xa inhibitors, such as rivaroxaban, apixaban, and edoxaban [18, 19]. However, the role of andexanet alfa in TBI hemorrhages is unclear, and its benefits must be weighed against the risk of thromboembolic complications [20, 21]. Andexanet alfa is a recombinant modified human factor Xa protein. It acts as a decoy protein for factor Xa inhibitors by binding to the anticoagulant drugs, neutralizing their activity, and restoring the ability of factor Xa to participate in the coagulation cascade [22].

The study addresses the lack of experimental data on andexanet alfa in the management of TBI under rivaroxaban anticoagulation, leveraging preclinical models to explore a potential benefit on hemorrhage reduction and overall improved outcomes.

## Methods

### Mice and Pretreatment

Male C57BL6/J mice (Janvier Labs) aged 8–12 weeks with a mean body weight of 25.8 ± 0.4 g were used. Mice were pretreated orally with rivaroxaban (10 mg/kg or 30 mg/kg body weight) 1 h prior the surgeries. These doses were chosen on the basis of previously established.

protocols showing effective anticoagulation [23–25]. All experiments were prospectively approved by the research ethics committee and continuously monitored (Regierungspraesidium Darmstadt, approval number FU/2054). Mice were housed under a 12 h light/dark cycle, held in groups of up to five animals per cage, and received water and food ad libitum. All surgery was performed under 2% isoflurane anesthesia and buprenorphine analgesia (10 mg/kg body weight). We conducted the study under consideration of the Animal Research: Reporting In Vivo Experiments (ARRIVE) guidelines [26]. All staff was trained in animal care (experimental animal science certificate FELASA-B).

### Hemostatic Efficacy

Rivaroxaban induced anticoagulation and hemostatic efficacy of andexanet alfa was evaluated using standard tail vein bleeding times (TVBT). For TVBT, 50 ml Falcon tubes were filled with saline and kept at 37 °C in a temperature-controlled water bath. The mice were anesthetized with a solution of ketamine (120 mg/kg) and xylazine (16 mg/kg) i.p. and placed over the Falcon tubes with the extended tail immersed in the prewarmed solution for 1 min. The tip of the tail was then cut 5 mm from the distal end. The tail was then immediately immersed in the prewarmed solution again. The TVBT is defined as the interval from cutting the tail to the end of the bleeding from the tip. If the bleeding time exceeded 20 min, the measurements were stopped and the TVBT was recorded as > 20 min.

### Study Design

**Fig. 1:**
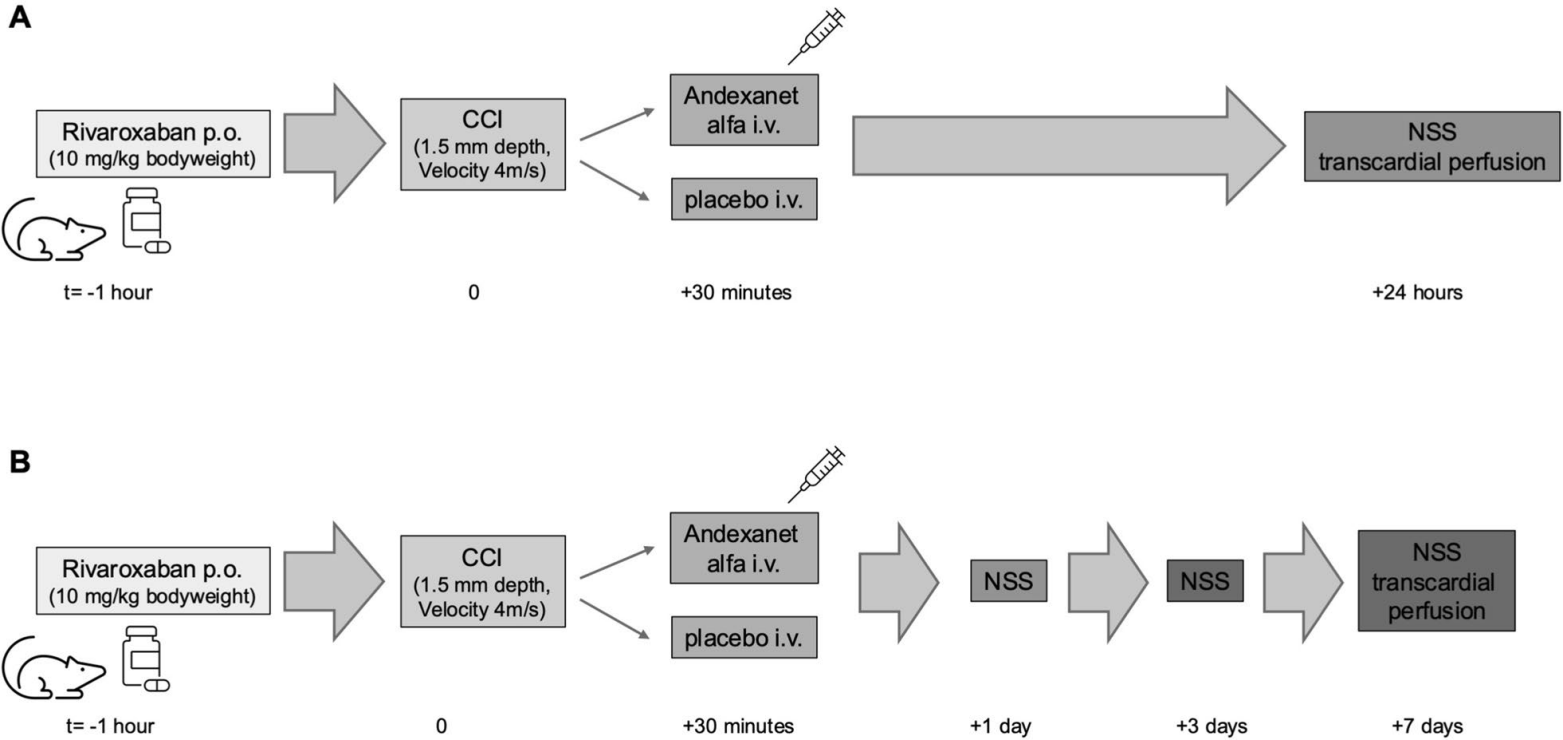
Timeline diagram of the experimental design. **A** Short-term observation period over 24 h. **B** Longer-term observation period over 7 days postinjury with more comprehensive functional testing

The main study consisted of two separate parts, I. a short-term experiment with termination after 24 h and II. a long-term observation over 1 week to assess the subacute stage as well (Fig. 1).

I. This part was designed to compare hemorrhage volumes and functional outcome in rivaroxaban pretreated mice 24 h after controlled cortical impact (CCI) between andexanet alfa and placebo-treated control mice. Andexanet alfa (0.96 mg 5 ml/kg bodyweight) was administered intravenously (i.v.) 30 min after the impact and compared with placebo-treated mice (phosphate-buffered saline (PBS, 5 ml/kg body weight), administered likewise i.v. 30 min after the impact).
II. The second part of our study consisted of a replication of the first part, but mice were then followed up for 7 days. Neurological outcomes were determined at day 1, 3, and 7 after CCI.

The operators performing surgical procedures (CCI and injections) and behavior tests, and the investigators evaluating data were blinded to the treatment groups. Based on the effect size of our previous studies (Cohens S = 1.75) [27], we calculated with at least 14 mice per group in order to detect a significant difference in hematoma volumes between treatment regimens with a power of 80% and a type 1 error of 0.05 using a two-tailed student’s *t*-test.

### Controlled Cortical Impact (CCI)

Mice were anesthetized with isoflurane and positioned in a stereotactic frame. A 5-mm craniotomy was made using a portable drill and a trephine over the right parietotemporal cortex, and the bone flap was removed. CCI was performed using a pneumatic cylinder with a 3-mm flat-tip impounder, 5 m/s velocity, 1.5 mm depth, and 150 ms impact duration. The severity of the traumatic brain injury can be considered moderate [28]. Subsequently, the bone flap was not replaced in order to limit the confounding effects of intracranial hypertension. All mice were then allowed to recover on a heating pad and were conscious and able to move after approximately 5 min.

### Neurological Severity Score (NSS)

In the first part of the study, functional deficits were assessed 24 h after CCI by using the Neurologic Severity Score (NSS). The NSS measures neurologic deficits on a 10-point ordinal scale [29], consisting of different motor, behavioral, and spontaneous locomotion tasks. In the second part of the study, the NSS was performed repetitively at day 1, 3 and 7 after CCI.

### Post-Mortem Magnetic Resonance Imaging (MRI)

After behavior assessment, deeply anesthetized mice were euthanized by transcardial perfusion with cold PBS (20 mL), followed by fixation using 4% paraformaldehyde (PFA, 20 ml). The mice heads were removed from the body and immersed in 4% PFA for 24 h and transferred to PBS for storage at 4 °C. MRI scanning was performed on a 7 Tesla small animal scanner (PharmaScan, Bruker, Ettlingen, Germany) equipped with a volume coil. Data acquisition was managed using the Paravision 6.0.1 software (Bruker, Billerica, MA, USA). Data analysis involved a blinded rater who quantified hematoma and edema volumes on T1- and T2-weighted images using Horos V 3.3.6 software (www.horosproject.org). Hemorrhages were directly quantified using manual volumetry. We used the indirect Kaplan method to calculate the extent of edema [30, 31]. Brain edema was expressed as a percentage of the contralateral unaffected hemisphere (extent of edema = (the volume of ipsilateral hemisphere–the volume of contralateral hemisphere)/the volume of contralateral hemisphere). Artifactual negative values arising from tissue loss in the absence of edema were constrained to zero for analysis.

### Histopathological Evaluations

After MRI scans, mice brains were stored in PBS at 4 °C and embedded in paraffin. The brains were sectioned at the impact site, which was visible on the brain surface, and then systematically at 1-mm intervals both anterior and posterior to this plane. Using a microtome (Quintessential Stereotaxic Injector, Stoelting), 3-µm sections were prepared, placed on SuperFrost Plus slides, and subsequently deparaffinized. Hematoxylin and eosin (HE) staining was performed, and after incubation, the slides were mounted in isopropanol followed by xylene. Evaluation and photographic documentation of the stained slides were performed using an Olympus BX-50 light microscope (Hamburg, Germany). Ordinal histoscores were calculated using a semi-quantitative assessment for hemorrhages and edema formation (For detailed description of the scoring system see Supplementary Materials).

### Statistics

Data was statistically analyzed using GraphPad Prism Version 10.4.1. Ordinal and non-normal continuous data is depicted as median and corresponding interquartile range (IQR), normally distributed continuous data is depicted as mean ± standard error of the mean (SEM) or standard deviation of the mean (SD). Data was assessed for normal distribution using the Kolmogorov–Smirnov-test and intergroup differences determined by *t*-test or Mann–Whitney-*U*-test, depending on the level of measurement. The significance level for all tests was set at *p* < 0.05.

## Results

### Andexanet alfa Reduced TVBT in Rivaroxaban Pretreated Mice

**Fig. 2:**
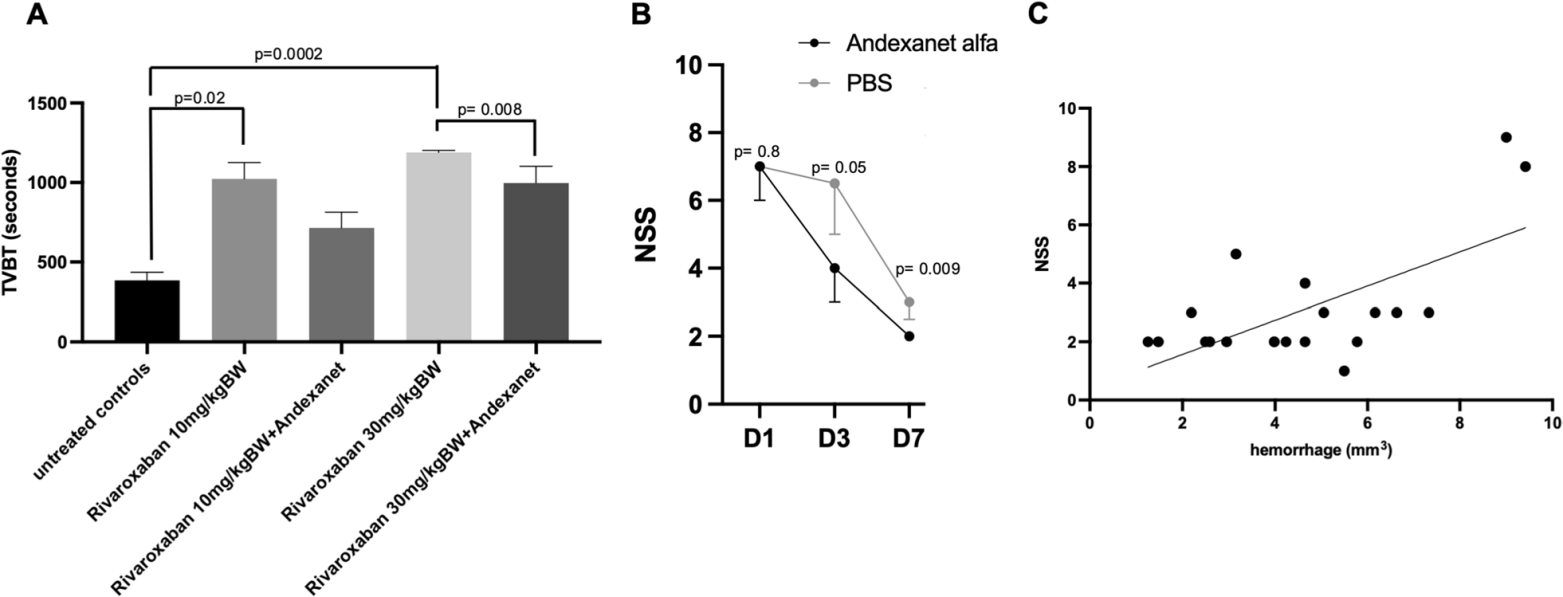
**A** Tail vein bleeding times (TVBT): Pretreatment with rivaroxaban increased TVBT (by 689 s at a dosage of 10 mg/kg bodyweight, *p* = 0.02; and by 853 s at a dosage of 30 mg/kg bodyweight, *p* = 0.0002). Increased TVBT could be reversed by application of andexanet alfa (by −212 s at a rivaroxaban dosage of 10 mg/kg bodyweight, *p* = 0.05; and by 95.0 s at a rivaroxaban dosage of 30 mg/kg bodyweight, *p* = 0.008). **B** Neurological Severity Scores (NSS): NSS scores did not differ at 24 h (median andexanet alfa = 7 (IQR 6–7); median placebo = 7 (IQR 6–7), *p* = 0.824) or at 3 days post-CCI (median ndexanet alfa = 4 (IQR 3–7); median placebo = 7 (IQR 5–8), *p* = 0.052). After 7 days, NSS scores were significantly different between groups (median andexanet alfa = 2 (IQR 2–2); median placebo = 3 (IQR 3–5), *p* = 0.0092). **C** Hematoma volume and functional outcome: Hemorrhage volumes strongly correlated with NSS scores at 7 days after CCI (*r* = 0.7, *p* = 0.002)

Given the varying protocols in the literature using rivaroxaban at doses of 10 or 30 mg/kg bodyweight [23–25], the first experiment aimed to determine an appropriate dose for subsequent experiments on the basis of functional effects assessed through standard TVBT. Anticoagulation with rivaroxaban at both dosages (10 and 30 mg/kg bodyweight) significantly increased TVBT compared with untreated control mice by a median of 689 s at a dosage of 10 mg/kg bodyweight (*p* = 0.02) and by a median of 853 s at a dosage of 30 mg/kg bodyweight (*p* = 0.0002, Fig. 2A). In detail, untreated controls displayed a median TVBT of 353 (IQR 257–252, *n* = 11) s. Mice pretreated with rivaroxaban 10 mg/kg bodyweight had a median TVBT of 1042 (IQR 835.5–1200, *n* = 5) s, after treatment with andexanet alpha the median was 830 (IQR 485–886.5, *n* = 5) s (*p* = 0.06). Animals treated with 30 mg/kg bodyweight had a median TVBT of 1200 (IQR 1169–1200, *n* = 5) s, after treatment with andexanet alpha the median was 1105 (IQR 820–1119, *n* = 5) s (*p* = 0.008, Fig. 2A). Taking these results together, we used rivaroxaban at a dose of 10 mg/kg bodyweight for the subsequent studies, as using the lower dose resulted in a larger effect size with less variability and surgical feasibility for the following craniotomies remained guaranteed.

### Neurological Outcome Improved in Andexanet Alfa-Treated Mice at Day 7

In the short-term experiment, we assigned 14 mice per group, of whom all survived and NSS scores were similar between andexanet alfa-treated and placebo-treated mice (median andexanet alfa = 6 (IQR 5–7), Median PBS = 6 (IQR 5–7), *p* = 0.9, *n* = 14/group).

Based on prior internal experience, we anticipated an elevated mortality rate over the course of the long-term study. To account for this, we included a reserve of 2 animals per group, resulting in an initial allocation of 16 animals per group. However, one mouse assigned to the placebo group died during surgery due to a complication of anesthesia. Consequently, the final group sizes were 16 mice for the Andexanet alfa treatment and 15 mice for the placebo group. Overall mortality in the model was 35.5%, and mortality rates (5/16 mice in the Andexanet alfa group and 6/15 mice in the placebo group) did not differ between groups (*p* = 0.72). There was no difference in neurological outcomes at either 24 h or 3 days (after 24 h: *p* = 0.824, median andexanet alfa = 7 (IQR 6–7), *n* = 15; median placebo = 7 (IQR 6–7), *n* = 14; at 3 days: *p* = 0.052, median andexanet alfa = 4 (IQR 3–7), *n* = 14; median placebo = 7 (IQR 5–8), *n* = 14). However, after 7 days, a significant difference in the neurological deficit could be demonstrated (*p* = 0.0092, median andexanet alfa = 2 (IQR 2–2), *n* = 11; median placebo = 3 (IQR 3–5), *n* = 9, Fig. 2B).

In our primary analysis, mice with severe neurological impairment were assigned a NSS of 10, while deceased animals were excluded from functional assessment, and their mortality was reported separately. We additionally performed a sensitivity analysis including deceased animals and assigning them a maximum NSS score of 10; the results of this analysis are presented in the Supplementary Material.

### Andexanet alfa Led to Reduced Hemorrhage Volumes

**Fig. 3:**
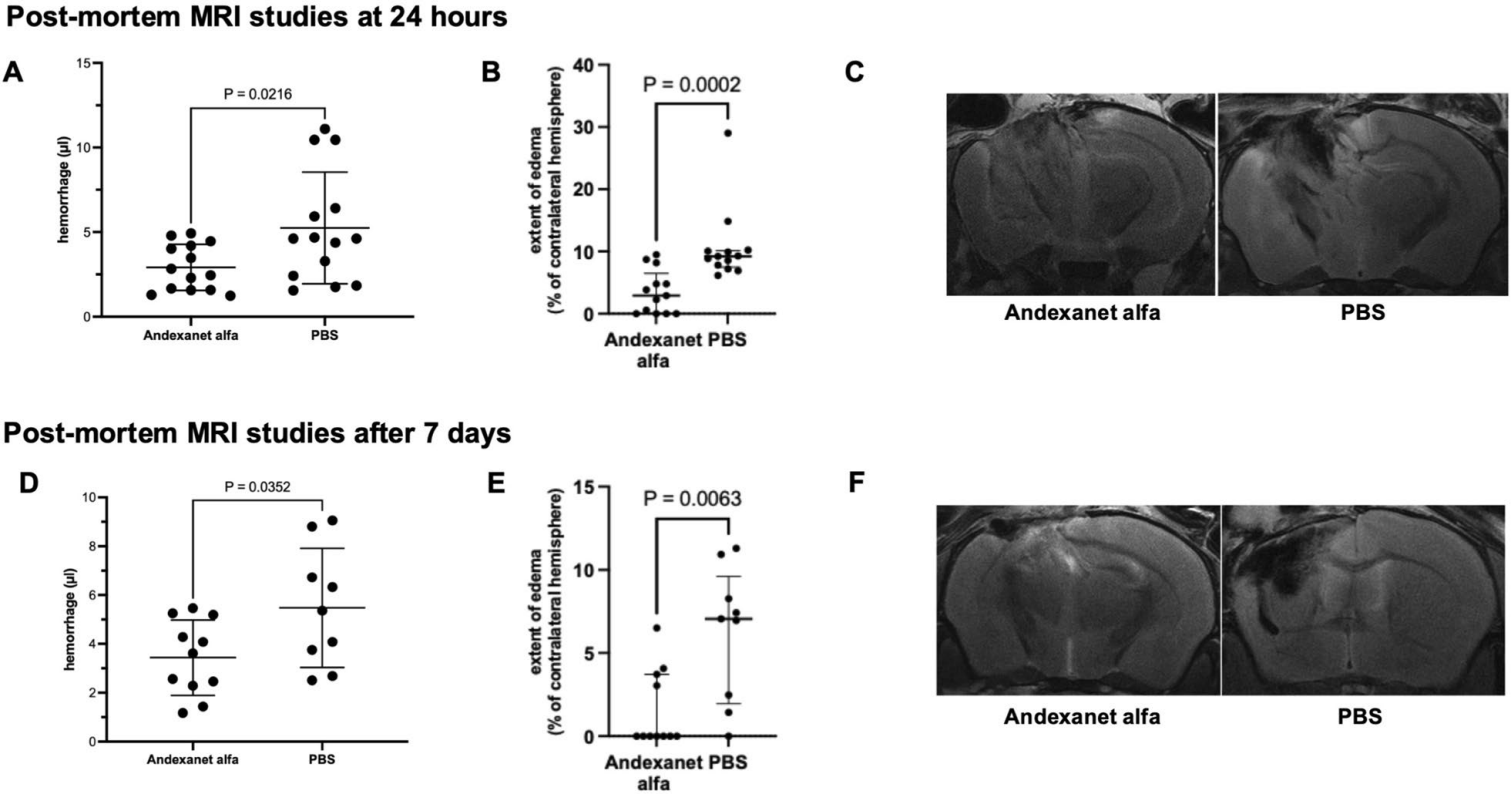
Hematoma and edema volumes measured by post-mortem MRI. **A** Hematoma sizes 24 h after CCI: Andexanet alfa reduced hemorrhage volume as compared with control group treated with PBS (2.9 ± 1.4 μl vs. 5.2 ± 3.3 μl, *n* = 14/group, *p* = 0.02). **B** Extent of edema (given as % of contralateral hemisphere) at 24 h after CCI: andexanet alfa reduced the extent of edema significantly (andexanet alfa median 2.9% (IQR 0–6.5%), PBS median 9.2% (IQR 7.5–10.1%), *p* = 0.0002). **C** Exemplary MRI images: post-mortem MRI slides of mice treated with andexanet alfa (left), and mice treated with placebo (PBS, right), sacrificed at 24 h postinjury. **D** Hematoma sizes 7 days after CCI: Hemorrhage volumes were significantly less in mice treated with andexanet alfa compared with control mice (3.4 ± 1.5 µl, *n* = 11 andexanet alfa treated mice vs. 5.5 ± 2.4 µl, *n* = 9, placebo treated mice; *p* = 0.04). **E** Extent of edema (given as % of contralateral hemisphere) 7 days postinjury: Andexanet alfa median 0% (IQR 0–3.7%), PBS median 7.1% (IQR 2.0–9.6%), *p* = 0.006. **F** Exemplary MRI images: post-mortem MRI slides of mice treated with andexanet alfa (left) and mice treated with placebo (PBS, right), sacrificed 7 days postinjury

Andexanet alfa reduced post-mortem MRI hematoma expansion 24 h after TBI as compared with the control group without reversal of anticoagulation (2.9 ± 1.4 μl vs. 5.2 ± 3.3 μl, *n* = 14/group, *p* = 0.02, Fig. 3A + C). The same applied to the bleeding volume in the longer-term trial after 7 days (3.4 ± 1.5 µl, *n* = 11 andexanet alfa treated mice vs. 5.5 ± 2.4 µl, *n* = 9, placebo treated mice; *p* = 0.04, Fig. 3D + F). Hemorrhage volumes strongly correlated with functional outcomes as measured by NSS scores (at 24 h post-CCI: *r* = 0.52, *p* = 0.007, *n* = 28; 7 days after CCI: *r* = 0.7, *p* = 0.002, *n* = 20, Fig. 2C) and with edema volumes (at 24 h post-CCI: *r* = 0.5, *p* = 0.02, *n* = 28; 7 days after CCI: *r* = 0.7, *p* = 0.001, *n* = 20, graphs not shown).

### Andexanet Alfa Reduced Edema Volumes

Along with the reduced hematoma sizes, andexanet alfa caused decreased post-mortem MRI edema extent (given as % of the contralateral hemisphere) 24 h after TBI as compared with the control group treated with PBS (−6.3%, *n* = 13/group, *p* = 0.0002, Fig. 3B). The same applied to the edema volume in the longer-term trial after 7 days, where overall edema volumes were smaller and more varying (−7.1%, *n* = 11 andexanet alfa-treated mice vs. *n* = 9 placebo-treated mice; *p* = 0.006, Fig. 3E).

### No Differences in Microscopic Hemorrhage or Edema Morphology

The explorative statistical analysis of the semi-quantitative histological evaluations of the sections showed no significant differences between the two groups at both time points (Supplementary Table S1). This concerns both the histopathological extent of the hemorrhage and the extent of edema development.

## Discussion

In summary, our experimental data suggests that the use of andexanet alfa improves functional outcomes in TBI under anticoagulation with rivaroxaban by reduction of factor Xa inhibitor-associated hematoma expansion. This aligns with its mechanism of action in reversing anticoagulation and reducing the extent of bleeding (hemostatic efficacy) as a significant contributor to prognosis after TBI [32].

While the ANNEXA I-trial in nontraumatic ICH failed to demonstrate improved mRS scores despite improved hemostatic efficacy [20], in our study the neurological deficit improved in the mice at day 7 after TBI. However, the follow-up period (of only 30 days instead of the usual 3 months in stroke research) in the ANNEXA I-trial was relatively short, which possibly explains the lack of evidence here. In mice at postinjury day 7—in between the acute and chronic phase—observations usually align with improved neurological deficits but not being back at baseline [33], still demonstrating differences between groups. The mortality rate in the longer-term study part was the same for both groups and corresponded to the level usually observed for the selected model severity [28]. We observed a correlation between increased hemorrhage and edema volumes, both presumably contributing to the worse functional outcome seen in the placebo-treated control mice without anticoagulant-reversal. A finding that aligns with observations made in patients experiencing TBI [34].

While the reduction of hemorrhage expansion and edema volumes by andexanet alfa could be measured macroscopically by MRI both in the short- and long-term, microscopic hemorrhage and edema characteristics were not different. It should be noted that accurate volumetric quantification at the microscopic level is inherently constrained. However, the correlation between hematoma expansion and functional outcomes underlines the clinical significance of hemorrhage control in improving neurological outcomes. Correlating with the reduced hematoma expansion, a significantly better neurological outcome was measured after 7 days. The fact that the neurological deficit did not differ on day 1 and day 3 is not related to bleeding-related effects, but to recovery after the operation, suggesting that andexanet alfa’s impact on functional recovery may require time to manifest, possibly owing to its effect on secondary injury mechanisms [35, 36].

Our work has several limitations. The studies focused solely on the acute and subacute phases, as these are clinically critical periods where neurological deterioration and complications such as hematoma expansion, seizures, and increased intracranial pressure significantly affect survival [37]. We excluded animals that did not survive from the primary analysis of functional outcomes, and we acknowledge that this limits the direct generalizability of functional outcome measures. However, this is an inherent limitation of preclinical outcome studies, as behavioral assessments cannot be performed in nonsurviving animals. Our intent was to specifically evaluate neurological outcomes in animals that survived, to assess recovery and treatment effects beyond survival alone. A further limitation is that andexanet alfa—different from its use in patients—was only administered as a bolus. Sufficient antagonization was nevertheless functionally demonstrated in the reduced TVBT and is in line with previous studies in mice [38]. However, the half-life of andexanet alfa in mice is 10–12 min according to internal data and was applied 30 min after TBI in our experimental setup. Whether a benefit is also present if andexanet alfa is applied at a greater time interval from the trauma is not answered by our experimental setup. In addition, the experiments were conducted exclusively on young male mice to minimize potential confounding factors associated with hormonal fluctuations and the potential prothrombotic properties of estrogen [39, 40]. Data on female and older mice, as well as on models incorporating comorbidities such as arterial hypertension or diabetes, remain limited. However, the existing literature on rivaroxaban does not point to relevant differences due to age or sex [41, 42]. Further studies are required to analyze these relevant subgroups. Our data could form a basis for this. Our study lacks sham surgeries. This is a concern due to potential confounding effects of rivaroxaban and/or andexanet alfa on secondary injury mechanisms as suggested by others [24, 35, 36], and about which we thus cannot provide any insights. Furthermore, the findings have not yet been extended to other mechanisms of injury (e.g., blunt vs. penetrating trauma). However, this simplified approach was deliberately chosen to provide a reliable and standardized framework that minimizes the influence of external variables, such as age, sex, injury mechanism, or comorbid conditions. Lastly, the lack of histological differences besides the proven reduction in lesion sizes marks that the underlying mechanisms warrant further investigation. Furthermore, one limitation of our study is the absence of a sham group, which could make interpretation of the results more challenging, particularly since a craniotomy was performed. The decision to omit a sham group was made to minimize animal use.

## Conclusions

Andexanet alfa shows promise in reversing anticoagulation effects of rivaroxaban and improving outcomes after TBI.

## Supplementary Information

Below is the link to the electronic supplementary material.

Supplementary file1 (DOCX 420 KB)
