# Supplementary material for: Andexanet alfa Reduces Hematoma Expansion Following Controlled Cortical Impact in Mice Pretreated with Rivaroxaban
Source: Neurocrit Care. 2026 Mar 3;45(1):200–7. doi: 10.1007/s12028-026-02463-w (PMC13369632; doi:10.1007/s12028-026-02463-w)
Supplement: Supplementary file 1 — Supplementary file1 (DOCX 420 KB) [file 12028_2026_2463_MOESM1_ESM.docx]

**Supplemental material:**

***Andexanet alfa reduces hematoma expansion following controlled cortical impact in mice*** ***pretreated with rivaroxaban***

Franziska Lieschke, MD^1,2 *^; Sarah Gelhard, MD^1, *^; Michelle Rosenthal-Rueckeis, MD^1^; Christian Grefkes, MD^1^; Ferdinand O. Bohmann, MD^1^

^1^Goethe University Frankfurt, University Hospital, Department of Neurology, Frankfurt/Main, Germany

^2^ Charité Universitätsmedizin Berlin, Department of Neurology with Experimental Neurology,

Berlin, Germany

*These authors contributed equally to this work and share first authorship

**Supplemental Methods:**

**Semiquantitative Assessment of Hemorrhage and Edema**

I. Hemorrhages:

HE staining, investigating the maximum impact within the slices and quantifying bleeding through the detection of extravascular erythrocytes as well as red-blood-cell-conglomerates on a microscopic 5-point scale:

0=no hemorrhage,

1=single petechial hemorrhage,

2=confluent petechial hemorrhage,

3=single space-occupying hemorrhage,

4=various, space-occupying hemorrhage.


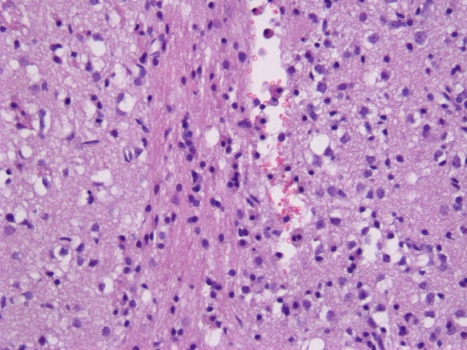

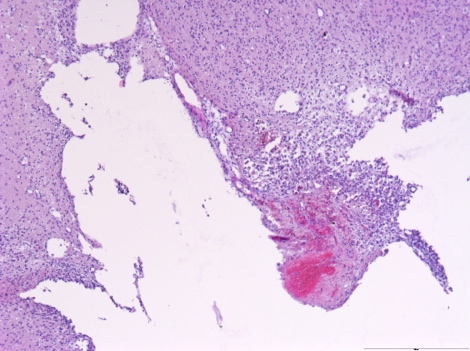

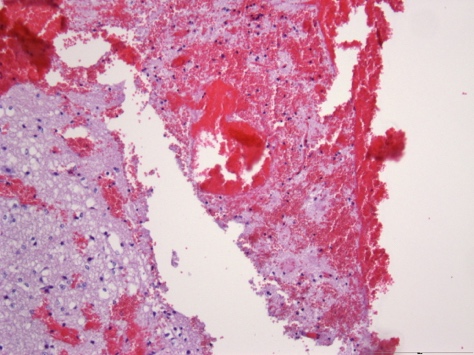


**Exemplary images:** left single petechial hemorrhage (Score:1), middle single space-occupying hemorrhage (Score: 3) and right various, space-occupying hemorrhage (Score: 4).

II. edema formation:

HE-stained sections, investigating the maximum impact within the slices analogous to I and quantifying edema through the detection loosening within the intercellular structure again on a 5-point scale:

0=no edema,

1=minimal edema,

2=minimal-medium edema and subcortical involvement,

3=medium-strong edema and subcortical involvement,

4=bilateral edema.

**Supplemental Results:**

**Supplemental Table S1**

| **Scores** | **Andexanet alfa** | **Placebo** | **p-value** |
| --- | --- | --- | --- |
| hemorrhages 24 hours | 4 (2-4) | 3 (2-3) | 0.3 |
| hemorrhages 7 days | 2 (2-3) | 3 (2-4) | 0.4 |
| edema 24 hours | 3 (2-4) | 2 (2-2) | 0.1 |
| edema 7 days | 2 (1.75-2.25) | 3 (2-4) | 0.2 |

**Table S1:** histological scores given as Median with interquartile range (IQR).

**Supplemental sensitivity Analysis**

When deceased mice were included and assigned an NSS score of 10, overall neurological outcomes showed improvement over time. While control mice initially exhibited a slight decline before recovering, those treated with Andexanet alfa demonstrated faster and greater recovery; however, these differences did not reach statistical significance.

At 24 hours: p=0.8, median andexanet alfa=7 (6-8), n=16; median placebo=7 (6-8), n=15;

at 3 days: p=0.16, median andexanet alfa=4 (3-8), n=16; median placebo= 8 (5-8), n=15;

and after 7 days: p=0.07, median andexanet alfa=2 (2-10), n=16; median placebo=5 (3-10), n=15.

Supplemental Figure S1. **Neurological Severity Scores (NSS)**
